# The Efficacy of Eculizumab in the Acute Phase of Neuromyelitis Optica Spectrum Disorder: A Case Series Study

**DOI:** 10.7759/cureus.73205

**Published:** 2024-11-07

**Authors:** Mitsuru Watanabe, Katsuhisa Masaki, Eizo Tanaka, Takuya Matsushita, Noriko Isobe

**Affiliations:** 1 Department of Neurology, Neurological Institute, Graduate School of Medical Sciences, Kyushu University, Fukuoka, JPN; 2 Department of Neurology, Kochi Medical School, Kochi University, Nankoku, JPN

**Keywords:** acute phase, attack, complement, eculizumab, neuromyelitis optica spectrum disorder, treatment

## Abstract

Eculizumab, a monoclonal antibody against complement C5, has been approved to prevent neuromyelitis optica spectrum disorder (NMOSD) relapse. Although complement activation leads to neuroinflammation and tissue necrosis in NMOSD attacks, clinical reports on the efficacy of eculizumab in the acute phase of NMOSD are limited. To clarify its effectiveness against clinical symptoms in NMOSD attacks, we described five patients with NMOSD who were administered eculizumab soon after treatment for an attack. The patients included four female patients and one male patient aged 50-93 years. The index attacks were optic neuritis in three cases, myelitis in one case, and brainstem encephalitis and myelitis in one case. Three patients had not received any maintenance therapy. Although all patients had received intravenous methylprednisolone (IVMP) and plasma exchange (PE) several times, these treatments were not sufficient to improve their symptoms. Thereafter, eculizumab was initiated between 35 and 61 days after the attack onset and partially ameliorated the symptoms in all cases. These cases suggest eculizumab as a treatment option to lessen the symptoms of severe NMOSD attacks, although the efficacy of IVMP and PE before eculizumab administration cannot be excluded.

## Introduction

Neuromyelitis optica spectrum disorder (NMOSD) is an inflammatory disorder of the central nervous system with pathological characteristics of preferential and massive astrocyte damage caused by antiaquaporin-4 antibody (AQP4-IgG) and complement activation [1,2]. Patients with NMOSD are sometimes disabled by severe attacks, regardless of extensive treatment with intravenous methylprednisolone (IVMP) and plasma exchange (PE). Therefore, it is important to prevent relapses, and additional treatment options to stop exacerbations and ameliorate symptoms are required. Eculizumab, a monoclonal antibody against complement C5, has been approved to prevent relapses of NMOSD [3]. Because complement activation causes neuroinflammation and tissue damage in attacks of NMOSD [2,4-6] and eculizumab reduces free C5 concentrations below the threshold for the complete inhibition of terminal complement within 60 minutes after the infusion [7], eculizumab is expected to be effective in the acute phase of the disease. However, few case reports have described the efficacy of eculizumab during the acute phase of NMOSD [8-11]. These include five patients for whom eculizumab effectively ameliorated the symptoms of NMOSD attacks, even after IVMP and PE had insufficient effects. Because all studies were case reports, further experiences and reports are needed to confirm the usefulness of eculizumab treatment during NMOSD attacks.

Between November 2019 and March 2022, we experienced five patients with AQP4-IgG-positive NMOSD in a clinical setting for whom eculizumab was administered soon after acute phase treatment for severe NMOSD attacks, and their symptoms were ameliorated after eculizumab initiation. Here, we describe these five cases in detail.

This article was previously presented as an ePoster at the 9th Joint European Committee for Treatment and Research in Multiple Sclerosis Americas Committee for Treatment and Research in Multiple Sclerosis Meeting (MSMilan2023), October 11-13, 2023.

## Case presentation

**Figure 1:**
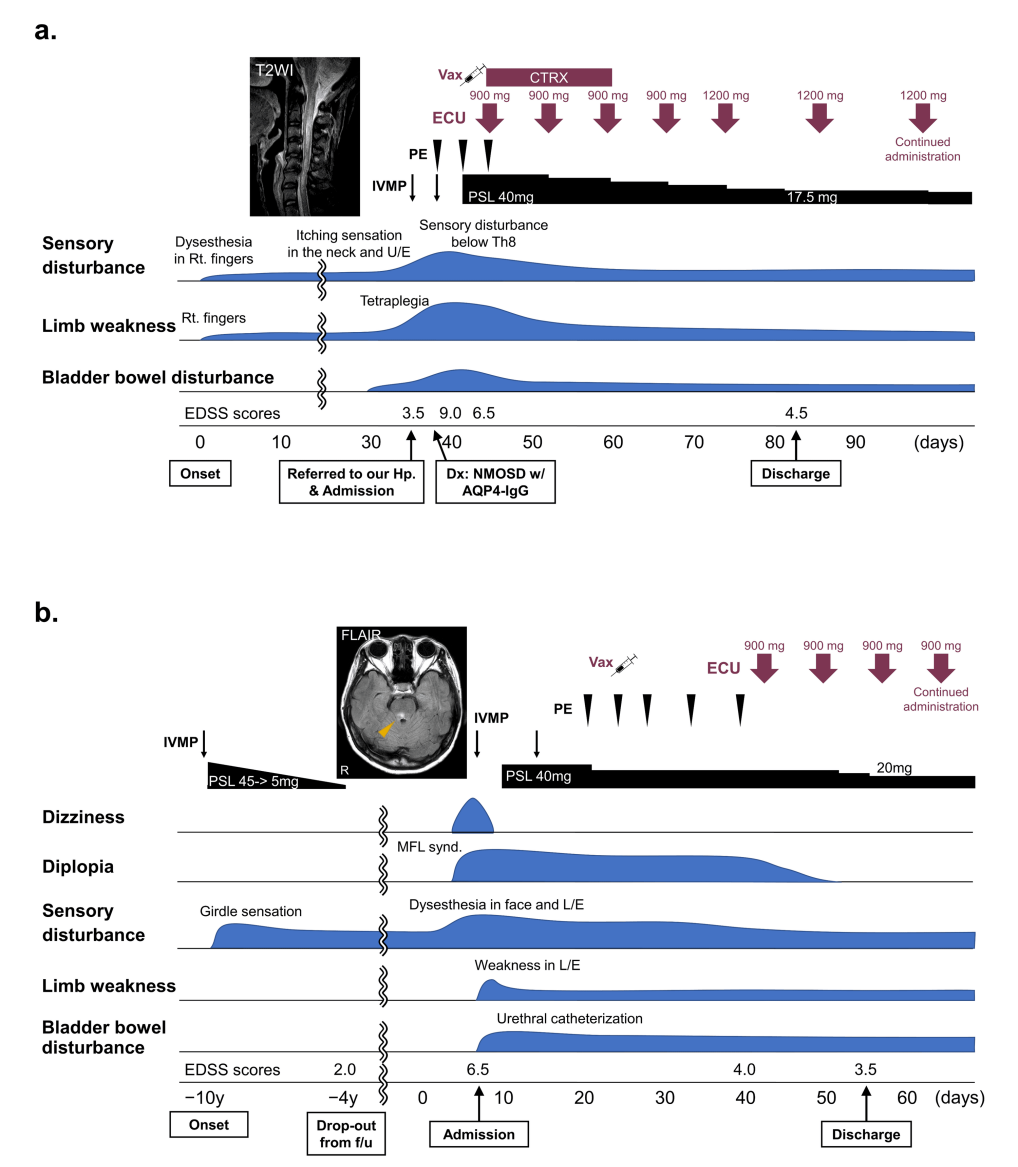
Clinical courses and treatment of cases 1 and 2 with spinal cord or brainstem lesions treated with ECU during the acute phase. (a) Case 1: a 50 y/o female patient with an MRI T2-weighted image showing longitudinal extensive transverse myelitis from C1 to C7 taken on day 34. (b) Case 2: a 53 y/o female patient with an MRI fluid-attenuated inversion recovery image showing a right-sided pontine tegmentum lesion (arrowhead). The onset day of the index attack was set as day 0 in each case AQP4-IgG: antiaquaporin-4 antibody; CTRX: ceftriaxone; Dx: diagnosis; ECU: eculizumab; EDSS: Expanded Disability Status Scale; f/u: follow-up; FLAIR: fluid-attenuated inversion recovery; Hp.: hospital; IVMP: intravenous methylprednisolone; L/E: lower extremity; Lt.: left; MFL: medial longitudinal fasciculus; NMOSD: neuromyelitis optica spectrum disorder; PE: plasma exchange; PSL: prednisolone; Rt.: right; T2WI: T2-weighted imaging; U/E: upper extremity; Vax: vaccination with meningococcal vaccine; w/: with; y/o: years old

**Figure 2:**
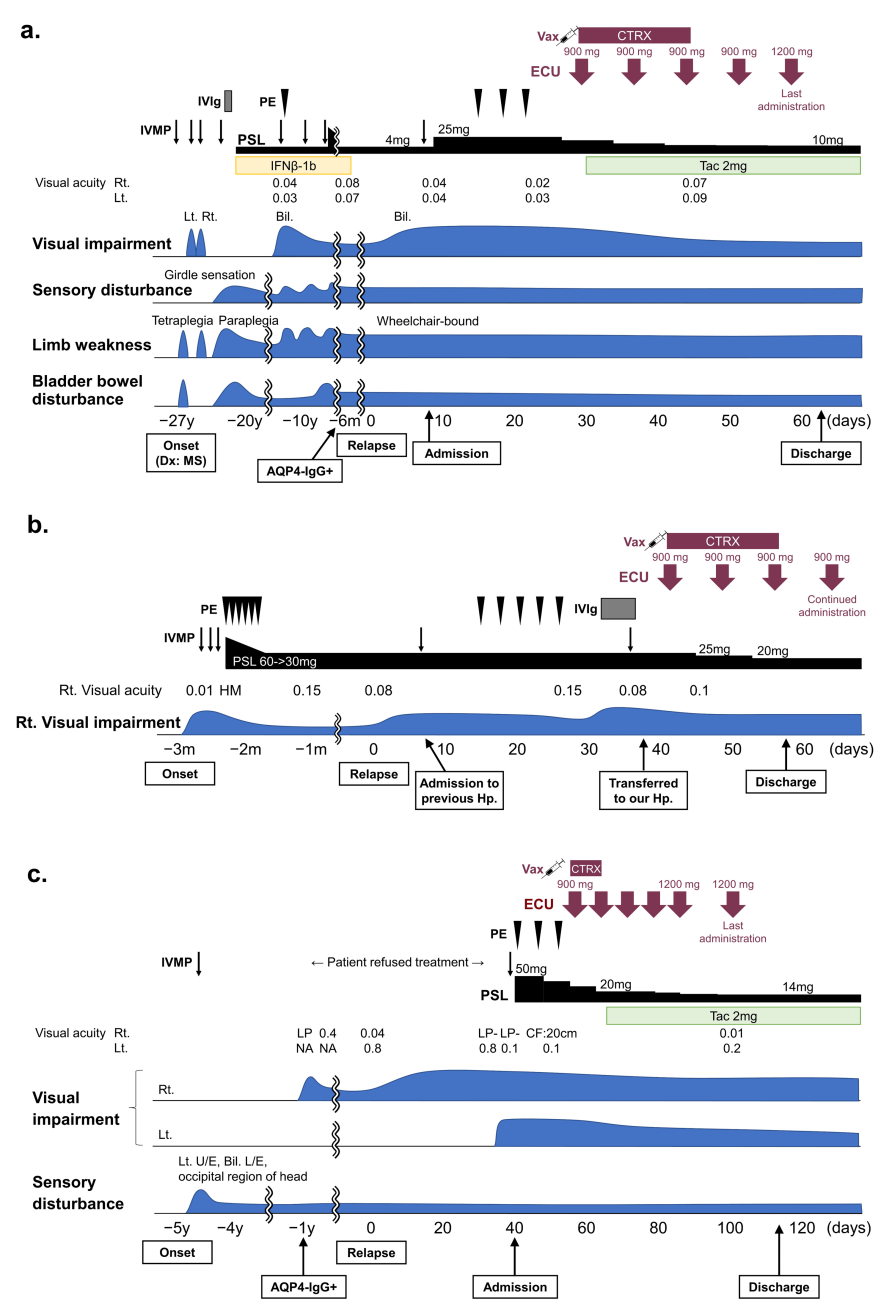
Clinical courses and treatment of cases 3-5 who experienced optic neuritis and were treated with ECU during the acute phase. (a) Case 3: a 78 y/o female patient. (b) Case 4: a 54 y/o female patient. (c) Case 5: a 93 y/o male patient. The onset day of the index attack was set as day 0 in each case AQP4-IgG: anti-aquaporin 4 antibody; Bil.: bilateral; CF: counting fingers; CTRX: ceftriaxone; Dx: diagnosis; ECU: eculizumab; HM: hand motion; Hp.: hospital; IFNβ: interferon β; IVIg: intravenous immunoglobulin; IVMP: intravenous methylprednisolone; L/E: lower extremity; LP: light perception; Lt.: left; MS: multiple sclerosis; NA: not applicable; PE: plasma exchange; PSL: prednisolone; Rt.: right; Tac: tacrolimus; U/E: upper extremity; Vax: vaccination of meningococcal vaccine; y/o: years old

Table 1 and Figures 1, 2 show the demographic and clinical characteristics and clinical courses of the cases.

**Table 1:** Demographic and clinical characteristics of the cases ^*^The following data are shown: Expanded Disability Status Scale (EDSS) scores and visual acuities of the eyes (Rt/Lt) ^a^The index attack was the onset of NMOSD ^b^Last EDSS score before dropout from the follow-up bil: bilateral; BS: brainstem; CF: counting fingers; ECU: eculizumab; IVIg: intravenous immunoglobulin; IVMP: intravenous methylprednisolone; LP-: no light perception; Lt: left; NA: not applicable; NMOSD: neuromyelitis optica spectrum disorder; ON: optic nerve; PE: plasma exchange; Rt: right; SC: spinal cord

Case	Sex	Age at onset	Age at onset of index attack	Lesions of the index attack	Treatment for the index attack before ECU initiation	Days from the onset of index attack to ECU initiation	Baseline disability before the index attack^*^	Worst disability during the index attack^*^	Disability at ECU initiation^*^	Disability after ECU treatment^*^
1^a^	Female	50	50	SC	IVMP and PE	46	0, NA/NA	9.0, NA/NA	6.5, NA/NA	4.5, NA/NA
2	Female	43	53	BS, SC	IVMP and PE	41	2.0,^b^ NA/NA	6.5, 1.2/1.2	4.0, 1.2/1.2	3.5, 1.2/1.2
3	Female	51	78	ON (bil)	IVMP and PE	30	7.5, 0.3/0.5	7.5, 0.05/0.05	7.5, 0.06/0.05	7.5, 0.4/0.3
4	Female	54	54	ON (Rt)	IVMP, PE, and IVIg	42	0.15/1.2	Severe visual field deficit/1.2	0.08/1.2	0.10/1.2
5	Male	88	93	ON (bil)	IVMP and PE	61	6.5, 0.8/0.9	6.5, LP-/0.1	6.5, CF (20 cm)/0.15	6.5, 0.01/0.2

Case 1

A 50-year-old woman developed dysesthesia and weakness in her right fingers, which was followed by a worsening and spread of the symptoms. She was referred and admitted to Kyushu University Hospital (Fukuoka, Japan) 35 days after onset with an Expanded Disability Status Scale (EDSS) score of 3.5. She was positive for AQP4-IgG, and spinal magnetic resonance imaging (MRI) showed longitudinal extensive transverse myelitis from C1 to C7. Even after two courses of IVMP and three courses of PE, her symptoms exacerbated to dysesthesia in both upper extremities, sensory disturbance below Th8 level, and tetraplegia with an EDSS score of 9.0; therefore, eculizumab was administered 11 days after admission just after meningococcal vaccination with a two-week intravenous infusion of ceftriaxone. Eculizumab treatment stopped the exacerbation of her symptoms, leading to their amelioration, and she was discharged from the hospital after 47 days of care, with an EDSS score of 4.5 (Figure 1a).

Case 2

A 53-year-old woman had a 10-year history of NMOSD with AQP4-IgG. Girdle sensation caused by myelitis was treated with IVMP, followed by oral corticosteroid tapering. Eight years after the disease onset, she dropped out from the follow-up. Two years later, she developed dizziness and diplopia because of medial longitudinal fasciculus syndrome, dysesthesia in her face and lower extremities, weakness in her lower extremities, and bladder and bowel disturbance requiring intermittent urethral catheterization. She was admitted to our hospital seven days after clinical relapse. An MRI of the brain and spinal cord detected brainstem and thoracic cord lesions. Although two courses of IVMP and five courses of PE ameliorated dizziness and weakness, diplopia remained. After meningococcal vaccination, eculizumab was started 34 days after admission, which improved the diplopia. She was discharged from our hospital with mild sensory disturbance, weakness in her lower extremities, and bladder dysfunction 48 days after admission (Figure 1b).

Case 3

A 78-year-old woman had a 27-year history of frequent attacks of myelitis and optic neuritis with severe disability and was wheelchair-bound (EDSS score 7.5). She had been diagnosed with multiple sclerosis and treated with oral corticosteroids and interferon-β. Half a year after she stopped interferon-β treatment because of AQP4-IgG positivity and a diagnosis as NMOSD with AQP4-IgG, she developed visual impairment in both eyes. One course of IVMP and three courses of PE failed to improve her visual function; therefore, 30 days after clinical relapse, eculizumab was administered in parallel with meningococcal vaccination, followed by a two-week intravenous infusion of ceftriaxone. Thereafter, her visual acuity improved gradually to the levels prior to the attack on her optic nerves. At the time of discharge, eculizumab had been stopped after the fifth infusion because of the patient's difficulty in continuing timely physician visits, and tacrolimus was administered to prevent future relapse (Figure 2a).

Case 4

A 54-year-old woman developed visual impairment to the hand motion level in her right eye and was treated with three courses of IVMP and six courses of PE. Despite this treatment, visual impairment with a visual acuity of 0.15 remained. She was diagnosed with NMOSD with AQP4-IgG, and oral corticosteroids were tapered following IVMP. Three months after treatment with 30 mg/day prednisolone, she developed visual impairment with a severe visual field deficit in her right eye. After one course of IVMP and five courses of PE, her optic neuritis was exacerbated, and she was transferred to our hospital 38 days after relapse, following another round of IVMP and intravenous immunoglobulin. Eculizumab was started four days after the transfer with meningococcal vaccination and concomitant initiation of a two-week intravenous infusion of ceftriaxone. Thereafter, her visual acuity mildly improved from 0.08 to 0.10. She was discharged from our hospital after 20 days and continued eculizumab treatment in our outpatient clinic (Figure 2b).

Case 5

A 93-year-old man had a history of myelitis five years previously, and when he developed right optic neuritis four years later, he was diagnosed with NMOSD with AQP4-IgG. One year after diagnosis, he relapsed with visual impairment in his right eye, for which he continuously refused to be treated. Around 35 days after the relapse of optic neuritis, he lost vision in his right eye and developed visual impairment in his left eye. After agreeing to treatment and hospital admission, he was treated with one course of IVMP and three courses of PE, which failed to ameliorate his visual function. Thereafter, he was vaccinated with meningococcal vaccine 11 days after admission and received eculizumab treatment 18 days after admission with one week of ceftriaxone treatment. Eculizumab was used safely and partially ameliorated his visual function. Before discharge, eculizumab was stopped because of difficulty in continuing timely physician visits, and tacrolimus was administered to prevent future relapse (Figure 2c).

No adverse events were observed in any of these cases. The suppression of complement activation after eculizumab initiation was confirmed by decreased CH-50 levels [12] in all cases, except case 5, in whom levels were not measured.

## Discussion

Here, we report five NMOSD cases for which eculizumab administration immediately after acute phase treatment improved their symptoms. To date, four articles have reported five cases in which eculizumab was used during the acute phase of NMOSD [8-11]. Our study includes the largest number of cases, and all their symptoms were improved by eculizumab treatment administered during the acute phase just after IVMP or PE. Our cases were in line with previously reported cases, all of which also showed improved disability after eculizumab treatment during the acute phase [8-11]. In the pathophysiology of NMOSD, the membrane attack complex damages central nervous system tissues and C5a attracts immune cells [5,13]. Furthermore, a recent study reported activated complement-induced Th17 cytokine bias in AQP4 autoimmunity [14]. Considering the important contribution of complement downstream and upstream in the pathogenesis of NMOSD and the rapid reduction of free C5 concentrations by eculizumab [7], the therapeutic effect of a C5 inhibitor during the acute phase of NMOSD might be expected. We also found good tolerability to eculizumab combined with two weeks of ceftriaxone just after meningococcal vaccination without actual meningococcal infection and fluctuation of the disease, even in an extremely elderly patient.

## Conclusions

Our cases suggest that eculizumab, a C5 inhibitor, might be a treatment option to ameliorate the symptoms of severe NMOSD attacks if disability improvement is insufficient or disease activity fluctuates, even after IVMP and PE treatment. We cannot exclude that the improved symptoms were related to the delayed effects of IVMP and PE use before eculizumab administration. Therefore, the exact effect of eculizumab during the acute phase should be confirmed in a case-control study with a large sample size or a randomized controlled trial. However, considering that patients with NMOSD are sometimes disabled by severe attacks, our case report sheds light on a new treatment strategy using eculizumab for NMOSD attacks to preserve patients' physical functions. Additionally, our cases show that eculizumab can be safely used within two weeks after meningococcal vaccination with concomitant use of ceftriaxone.
